# RETRACTION: Effect of Chinese herbal compound dressings in treating patients with diabetic foot ulcers: A meta‐analysis

**DOI:** 10.1111/iwj.70077

**Published:** 2024-09-24

## Abstract

Retraction: YangQ. , LiuF. , ZhaoC. , XuX. , WangY. , ZuoW. , “Effect of Chinese Herbal Compound Dressings in Treating Patients with Diabetic Foot Ulcers: A Meta‐Analysis,” International Wound Journal 21, no. 3 (2023): e14767, 10.1111/iwj.14767.PMC1091470938444012

The above article, published online on 05 March 2024, in Wiley Online Library (http://onlinelibrary.wiley.com/), has been retracted by agreement between the journal Editor in Chief, Professor Keith Harding; and John Wiley & Sons, Ltd. It came to the publisher's attention from a third party that a number of articles shared concerning similarities in format and structure. Following an investigation by the publisher, the retraction has been agreed on as the peer review and publishing process for this article were found to be manipulated.

Retraction: Q. Yang , F. Liu , C. Zhao , X. Xu , Y. Wang , W. Zuo , “Effect of Chinese Herbal Compound Dressings in Treating Patients with Diabetic Foot Ulcers: A Meta‐Analysis,” International Wound Journal 21, no. 3 (2023): e14767, 10.1111/iwj.14767.PMC1091470938444012

The above article, published online on 05 March 2024, in Wiley Online Library (http://onlinelibrary.wiley.com/), has been retracted by agreement between the journal Editor in Chief, Professor Keith Harding; and John Wiley & Sons, Ltd. It came to the publisher's attention from a third party that a number of articles shared concerning similarities in format and structure. Following an investigation by the publisher, the retraction has been agreed on as the peer review and publishing process for this article were found to be manipulated.

